# Diagnosing Crohn’s disease in presumed cryptoglandular perianal fistulas: an expert Delphi consensus on early identification of patients at risk of Crohn’s disease in perianal fistulas (PREFAB)

**DOI:** 10.1093/ecco-jcc/jjaf002

**Published:** 2025-01-07

**Authors:** Liesbeth J Munster, Luke N Hanna, Ailsa L Hart, Phil J Tozer, Christianne J Buskens, Jarmila D W van der Bilt, TOpCLASS perianal Crohn’s disease Consortium, the Dutch Initiative on Crohn and Colitis (ICC) and the Initiative on Crohn and Colitis - Surgery (ICC-S), Anders Dige, Lilli Lundby, Christianne J Buskens, Jaap Stoker, Jarmila D W van der Bilt, Dermot P B McGovern, Benjamin L Cohen, Stefan D Holubar, Nick Powell, Shaji Sebastian, Antonino Spinelli, Michele Carvello, Serre-Yu Wong, Jean-Frédéric Colombel, Ignacio Catalán-Serra, Susan J Connor, Sulak Anandabaskaran, Jean-Frédéric Leblanc, Amy L Lightner, Ailsa L Hart, Phil J Tozer, Kapil Sahnan, Philip F C Lung, Nik S Ding, Corina Behrenbruch, Leon S Winata, Jeffrey D McCurdy, Jeroen Geldof, Danny De Looze, Isabelle De Kock, Séverine Vermeire, Bram Verstockt, André D’Hoore, Gabriele Bislenghi, David T Rubin, Benjamin D McDonald, Parakkal Deepak, David H Ballard, Paulo G Kotze, Carla B Harmath, Sara El Ouali, Laurents P S Stassen, Froukje J Hoogeboom, Marijn C Visschedijk, Koen W van Dongen, Marjolijn Duijvestein, Oddeke van Ruler, Koen C M J Peeters, Andrea E van der Meulen-de Jong, Milan C Richir, Fiona D M van Schaik, Marie J Pierik, Marco W Mundt, David D E Zimmerman, Ingrid J M Han-Geurts

**Affiliations:** Department of Surgery, Flevoziekenhuis, Almere, The Netherlands; Department of Surgery, Amsterdam UMC, location VUmc, Amsterdam, The Netherlands; Department of Gastroenterology and Hepatology, St. Mark’s Hospital, London, United Kingdom; Department of Gastroenterology and Hepatology, St. Mark’s Hospital, London, United Kingdom; Department of Surgery, St. Mark’s Hospital, London, United Kingdom; Department of Surgery, Amsterdam UMC, location VUmc, Amsterdam, The Netherlands; Department of Surgery, Flevoziekenhuis, Almere, The Netherlands; Department of Surgery, Amsterdam UMC, location VUmc, Amsterdam, The Netherlands; Department of Hepatology and Gastroenterology, Aarhus University Hospital, Denmark; Department of Surgery, Aarhus University Hospital, Denmark; Department of Surgery, Amsterdam UMC, location VUmc, Amsterdam, The Netherlands; Department of Radiology, Amsterdam UMC, The Netherlands; University of Amsterdam, The Netherlands; Department of Surgery, Flevoziekenhuis, Almere, The Netherlands; Department of Surgery, Amsterdam UMC, location VUmc, Amsterdam, The Netherlands; F. Widjaja Inflammatory Bowel Disease Institute, Cedars-Sinai Medical Center, Los Angeles, CA, USA; Department of Gastroenterology, Hepatology, and Nutrition, Digestive Disease Institute, Cleveland Clinic, Ohio, USA; Department of Colorectal Surgery, Digestive Diseases Institute, Cleveland Clinic, Ohio, USA; Department of Digestion, Metabolism and Reproduction, Faculty of Medicine, Imperial College London, UK; IBD Unit, Department of Gastroenterology, Hull University Hospitals NHS Trust, Hull, UK; Department of Biomedical Sciences, Humanitas University, Pieve Emanuele, Milan, Italy; IRCCS Humanitas Research Hospital, Rozzano, Milan, Italy; Humanitas Clinical and Research Hospital, Rozzano, Milan, Italy; The Dr Henry D. Janowitz Division of Gastroenterology, Icahn School of Medicine at Mount Sinai, New York, NY, USA; The Dr Henry D. Janowitz Division of Gastroenterology, Icahn School of Medicine at Mount Sinai, New York, NY, USA; Centre of Molecular Inflammation Research, Norwegian University of Science and Technology, Norway; Department of Clinical and Molecular Medicine, Norwegian University of Science and Technology, Trondheim, Norway; Gastroenterology, Department of Medicine, Levanger Hospital, Nord-Trøndelag Hospital Trust, Levanger, Norway; The University of New South Wales, Sydney, Australia; Department of Gastroenterology, Liverpool Hospital, Sydney, Australia; The University of New South Wales, Sydney, Australia; Department of Gastroenterology, Nepean Hospital, Sydney, Australia; Montreal Sacred Heart Hospital, University of Montreal, Montreal, Canada; Molecular Medicine and Colorectal Surgeon, Scripps Research Institute, La Jolla, CA, USA; Department of Gastroenterology, St. Mark’s Hospital & Academic Institute, UK; Department of Surgery, St. Mark’s Hospital & Academic Institute, UK; Department of Surgery, St. Mark’s Hospital & Academic Institute, UK; Department of Radiology, St Mark’s Hospital & Academic Institute, UK; Department of Gastroenterology, St. Vincent’s Hospital Melbourne, Australia; Department of Surgery, St. Vincent’s Hospital Fitzroy Melbourne, Australia; Department of Radiology, St Vincent’s Hospital, Melbourne, Australia; Department of Medicine, Division of Gastroenterology, Ottawa, University of Ottawa, Canada; The Ottawa Hospital Research Institute, Ottawa, Canada; Department of Gastroenterology, University Hospital Ghent, Belgium; Department of Gastroenterology, University Hospital Ghent, Belgium; Department of Radiology, University Hospital Ghent, Belgium; Department of Gastroenterology and Hepatology, Leuven University Hospitals, Leuven, Belgium; Department of Chronic Diseases and Metabolism, KU Leuven, Translational Research Center for Gastrointestinal Disorders (TARGID), Leuven, Belgium; Department of Chronic Diseases and Metabolism, KU Leuven, Translational Research Center for Gastrointestinal Disorders (TARGID), Leuven, Belgium; Department of Gastroenterology and Hepatology, Leuven University Hospitals, Leuven, Belgium; Department of Abdominal Surgery, University Hospitals Leuven, Leuven, Belgium; Department of Abdominal Surgery, University Hospitals Leuven, Leuven, Belgium; University of Chicago Medicine Inflammatory Bowel Disease Center, Chicago, USA; University of Chicago Medicine Inflammatory Bowel Disease Center, Chicago, USA; Washington University School of Medicine in St. Louis, St. Louis, Missouri, USA; Mallinckrodt Institute of Radiology, Washington University St. Louis School of Medicine; St. Louis, Missouri, USA; Health Sciences Postgraduate Program, Pontificia Universidade Catolica do Parana (PUCPR), Curitiba, Brazil; University of Chicago Medicine Inflammatory Bowel Disease Center, Chicago, USA; Digestive Disease Institute, Cleveland Clinic Abu Dhabi, United Arab Emirates; Department of Surgery, Maastricht University Medical Center, The Netherlands; Nutrim Institute of Nutrition and Translational Research in Metabolism, Maastricht University, The Netherlands; Department of Surgery, University Medical Center Groningen, The Netherlands; Department of Gastroenterology and Hepatology, University Medical Centre, Groningen, The Netherlands; Department of Surgery, Maasziekenhuis Pantein, Boxmeer, The Netherlands; Department of Gastroenterology and Hepatology, Radboud University Medical Center, Nijmegen, The Netherlands; Department of Surgery, IJsselland Hospital, The Netherlands; Department of Surgery, Erasmus Medical Center, The Netherlands; Department of Surgery, Leiden University Medical Center; Department of Gastroenterology and Hepatology, Leiden University Medical Center, The Netherlands; Department of Surgery, University Medical Center Utrecht, The Netherlands; Department of Gastroenterology and Hepatology, University Medical Center Utrecht, The Netherlands; Department of Gastroenterology-Hepatology, School for Nutrition and Translational Research in Metabolism (NUTRIM), Maastricht University Medical Centre+, Maastricht, The Netherlands; Department of Gastroenterology and Hepatology, Flevoziekenhuis, Almere, The Netherlands; Fistula Expertise Center, ETZ, Tilburg, The Netherlands; Proctos Clinics, The Netherlands

**Keywords:** perianal fistula, perianal disease, Crohn’s disease

## Abstract

**Background:**

The aim of this Delphi study was to reach consensus on a new clinical decision tool to help identify or exclude Crohn’s disease (CD) in patients with perianal fistula(s) (PAF).

**Methods:**

A panel of international experts in the field of proctology/inflammatory bowel disease was invited to participate. In the first round (electronic survey), participants were asked to anonymously provide their opinion probing (1) the relevance and use of clinical characteristics suggestive of underlying CD, (2) the use of fecal calprotectin (FCP) for screening for CD, and (3) on the diagnostic work-up for CD in PAF patients with raised clinical suspicion. In the second/third round (virtual consensus meetings), statements were paired/revised and presented in final sets of statements. Consensus was predefined as ≥70% (dis)agreement.

**Results:**

Final consensus was reached on 12 statements, including screening of all PAF patients (regardless of the complexity, biological behavior, and co-existent perianal symptoms) and referral of PAF patients for a colonoscopy in case of elevated FCP levels (≥150 mcg/g) and/or in case of one clinical major criterion (defined as: unintentional weight loss, unexplained diarrhea, PSC, UC, >1 internal fistula openings, fistula involving other organs (vagina/bladder), recurrent fistulation (after initial healing), proctitis, and anal stenosis). Also, clinical (fistula-)characteristics that warrant raised suspicion for CD and an algorithm on the diagnostic work-/follow-up of patients with raised suspicion were defined.

**Conclusion:**

International consensus was reached on a new, clinical decision tool, including a practical and relevant algorithm for finding/excluding CD in PAF patients.

**Key Messages**

**- What is already known**

A prolonged time to Crohn’s disease (CD) diagnosis is common, especially in patients in whom a perianal fistula is the manifesting sign. This may result in progression of disease and worse outcomes.

**- What is new here**

International consensus was reached on a new, clinical decision tool, including a practical and relevant algorithm for identifying or excluding CD in patients with a perianal fistula as a manifesting sign.

**- How can this study help patient care**

This new, clinical decision tool is an easy-to-use tool that could serve as guidance to all healthcare professionals who interact with patients with perianal fistulas. By implementing this tool in daily clinical practice, it is assumed that the time to CD diagnosis as well as surgical/pharmaceutical treatment can be reduced, so that (perianal) CD can be identified at an early stage, and that (long-term) outcomes in (perianal) CD patients can be improved.

## 1. Introduction

Perianal fistulas (PAF) are the initial disease manifestation in approximately 10% of patient with Crohn’s disease (CD).^1–3^ Importantly, delays in the diagnosis of CD are common in this population. In a recent systematic review involving patients with PAF as the initial manifestation of CD, the weighted mean time to CD diagnosis was 45.9 (SD 31.3) months.^2–6^ It was also shown that a prolonged time to CD diagnosis in patients with a PAF as a manifesting sign is associated with worse long-term outcomes.^7^ This same study showed that patients whom achieved radiological healing, nowadays the best predictor for no recurrence, had the shortest time to CD diagnosis.^7^ In line with this, it was previously shown that progression of a simple PAF into a complex PAF is associated with higher proctectomy and stoma rates.^8^ Therefore, earlier identification and increased clinical awareness of CD in this patient population may allow for early, disease-specific intervention to reduce the risk of disease progression.

Several clinical characteristics, so-called “red flags,” for patients with PAF at risk of CD were identified in a recent systematic review and retrospective matched cohort study, including young age < 40 years, weight loss, abdominal pain, and >2 previous perianal interventions, with a ROC analysis showing that a combination of these red flags was associated with good discrimination of CD versus non-CD (AUC 0.83 [0.72-0.94]).^9^ It has also been described that several fistula characteristics (ie, multiple internal and external openings, fissures, and proctitis) could raise the suspicion of underlying CD. Finally, fecal calprotectin (FCP) has been suggested as a potential biomarker to discriminate perianal CD from cryptoglandular PAF, even in the absence of luminal inflammation (optimal cutoff of ≥150 mcg/g),^10^ with several studies showing that FCP is an efficient and cost-effective approach in identifying patients with underlying CD.^11–14^ However, clear guidelines for daily clinical practice on when and how to proceed to further diagnostics are currently lacking. Therefore, the aim of this study was to reach international consensus on a clinical decision tool to help identify patients at risk of CD when a PAF is the initial manifestation and thereby reduce the time to CD diagnosis and treatment.

## 2. Materials and methods

This Delphi study was conducted between May 2023 and July 2024 and was conducted in parallel with the isolated perianal Crohn’s disease (ipCD) Delphi consensus study, aiming at defining ipCD and to conduct novel diagnostic criteria and management options in these specific patients. An engaged steering committee was appointed (L.H., L.M., A.H., P.T., C.B., J.B.). This steering committee assessed the feasibility and framed the procedures during the first, second, and if needed third rounds of this study. A panel of international experts in the field of proctology and/or Inflammatory Bowel Disease, consisting of surgeons, gastroenterologists, and radiologists, were invited to participate in all parts of the study (see Acknowledgments for all expert panel members). Specific details of all participating expert panel members were collected (eg, age, gender, race, level of experience, whether they work at a specialist/non-specialist hospital setting and expertise regarding the topic (eg, publications, clinical experience)). Participation in this study was on a voluntary base. The first round consisted of an electronic survey in which participants were asked to anonymously provide their opinion probing (1) the relevance and use of clinical characteristics (“red flags”) suggestive of underlying CD, (2) the use of FCP as an adjunct for screening for CD and (3) on the diagnostic work-up for CD in PAF patients with raised clinical suspicion on having CD. This survey was constructed by the use of Google Forms (Google Inc. https://docs.google.com/forms/d/e/1FAIpQLSf7QaCIA9qEO5BRY0YGvY7VqNyHbP9GnCIdF0eF4unNP4n3sg/viewform?usp=sf_link) and was sent to all potential participants via an e-mail containing a link to the survey. This e-mail also contained a video to explain the purpose of this consensus project in order to give context to the questions that were asked. Also, a summary of relevant abstracts from the literature was attached. Written consent for the use of all input was implied at the time that the participant completed the survey. Questions were based on three-point Likert scales (ranging from “very suggestive” to “not suggestive”) or five-point Likert scales (ranging from “strongly agree” to “strongly disagree”). Predefined consensus was achieved in case ≥70% of all participants (strongly) agreed on the statement. Consensus not to include statements was also achieved in case ≥70% of all participants (strongly) disagreed on a statement. If applicable, the response option “other,” including free textboxes, was implemented in accordance with the standard Delphi methods. An opportunity for feedback/questions was also provided at the end of the survey by providing a free textbox.

All statements were paired/revised based on the feedback from the first round and presented in a final set of statements in a second round. In this second round, a virtual consensus meeting was conducted (www.teams.microsoft.com). At the beginning of this meeting, all participants were provided with background information based on all separate items in the survey. All statements from the first round were assessed. In case no consensus was reached in the first round, statements were merged and/or rephrased and interim votes were conducted until consensus ≥70% was reached for each separate statement. If needed, a third virtual meeting was initiated. Voting was accomplished by the use of Poll Everywhere (www.polleverywhere.com).

### 2.1. Ethics

This study did not fall within the scope of the Medical Research Involving Human Subjects Act (WMO).

### 2.2. Statistical analyses

Data were presented as the total amount of participants that agreed on all separate statements including the percentages of agreement. All analyses were performed by the use of SPSS Statistics for Windows (version 22, IBM Crop., Armonk, NY, USA).

## 3. Results

A total of 30 experts participated, with a median of 13 years of specialty experience (IQR 10-21). Consensus was reached on a total of 12 statements to be included in the clinical decision tool. An overview of all final statements is provided in Table 1.

**Table 1.** Final statements.

	Final statement	Consensus (%)
1.	Shortening the delay in CD diagnosis, by using a clinical decision tool, could improve outcomes in patients where perianal disease is their first symptom of underlying CD	97
2.	An index of high-risk features, associated with CD, in PAF patients, would be a useful tool to help clinicians identify patients who should be investigated for CD	97
3.	The following clinical (fistula) characteristics and/or MRI findings and/or US findings *(if already performed)* may be suggestive of pCD/warrant raised suspicion for CD in patients with PAF and should therefore be included in the clinical decision tool (see Table 2)	88
4.	FCP is an adjunct for raising suspicion of CD in PAF patients when there is clinical suspicion of CD	100
5.	FCP should be measured as an early risk stratification test for early identification of CD in patients with any perianal fistula without any red flags which would warrant investigation	100
6.	The optimal cutoff value of FCP as risk stratification tool is ≥150 mcg/g	92
7.	A colonoscopy for early identification of CD should be performed in PAF patients with elevated FCP levels (≥150 mcg/g) and/or clinical suspicion (see 8)	92
8.	A colonoscopy should be performed in case of one clinical major criterion, defined as: unintentional weight loss (72%), unexplained diarrhea (75%), PSC (72%), UC (78%), >1 internal fistula opening (88%), fistula that involves other organs (vagina/bladder, 88%), recurrent fistulation (after initial healing, 72%), proctitis (100%), and anal stenosis (88%)	94
9.	A colonoscopy should be based on current guidelines and therefore should include assessment of the terminal ileum and random biopsies of the ileum and colon	92
10.	If colonoscopy is normal and clinical suspicion remains, then patients should be referred for small bowel investigation (according to (local) guidelines)	100
11.	A patient can be diagnosed with a Crohn’s perianal fistula based on having: “any perianal fistula with associated luminal investigations showing radiological, endoscopic, or histologically confirmed luminal inflammation”	87
12.	Patients with a suspicion of CD in whom luminal disease has been excluded and cannot be categorized as isolated perianal CD should have standardized outpatient clinic follow-up in 1 year to re-assess the clinical decision tool for any new evidence on CD	88

CD, Crohn’s disease; FCP, fecal calprotectin; PAF, perianal fistula(s).

Ninety-seven percent of participants agreed that shortening of the delay in diagnosis by using a clinical decision tool could improve outcomes in patients with perianal disease as a first symptom. However, only 33% of all participants stated that they have a standardized protocol for referral of patients with PAF, suspected of having CD.

### 3.1. The relevance and use of clinical (fistula) characteristics (“red flags”) suggestive of underlying CD

The majority of participants (97%) agreed that an index of high-risk features, associated with CD, in PAF patients would be a useful tool to help clinicians identify patients who should be investigated for CD. In total, 40 clinical (fistula) features that should raise clinical suspicion for CD in patients presenting with a PAF were identified in the first and second rounds (see Table 2). Fistula characteristics were divided into complexity, biological behavior, and associated symptoms. It was agreed that fistula characteristics found on MRI and/or United States should also be included, but only if MRI and/or United States was already performed (eg, this should not delay your diagnostic work-/follow-up).

**Table 2.** Clinical (fistula) characteristics included in the clinical decision tool that may be suggestive of pCD/warrant raised suspicion of CD in patients with PAF.

The following clinical (fistula) characteristics (red flags) and/or MRI findings and/or US findings *(if already performed)* may be suggestive of pCD in patients with PAF:			
Clinical characteristics	Red flags	Round 1	Round 2
Demographic data	1. Age < 40 years	93%	N/A
Medical/family history	2. Family history	100%	N/A
	3. Smoking	77%	N/A
	4. Previous perianal interventions^a^	100%	N/A
	5. Previous bowel surgery^a^	90%	N/A
	6. History of abdominal pain in childhood	73%	N/A
Symptoms	7. Rectal bleeding	100%	N/A
	8. Unexplained abdominal pain	97%	N/A
	9. Unintentional weight loss	93%	N/A
	10. Unexplained diarrhea	93%	N/A
	11. Unexplained fatigue	87%	N/A
	12. Anemia	90%	N/A
	13. Vitamin deficiencies	87%	N/A
	14. Aphthous stomatitis	N/A	87%
Extraintestinal symptoms	15. Hidradenitis suppurativa	100%	100%
	16. Inflammatory arthritis	97%	100%
	17. Ankylosing spondylitis	96%	100%
	18. Psoriasis	83%	100%
	19. Erythema nodosum	97%	100%
	20. Uveitis	100%	100%
	21. PSC	N/A	100%
Other (misinterpreted) diagnosis related to bowel symptoms	22. Ulcerative colitis	90%	89%
	23. IBS	63%	89%
	24. Coeliac disease	50%	89%
	25. Lactose intolerance	37%	89%
	26. Eating disorder	37%	89%
Fistula characteristics			
Complexity of the fistula	27. >1 internal openings	100%	N/A
	28. >1 external openings	97%	N/A
	29. A complex (rather than simple) fistula	97%	N/A
	30. Fistula involves other organs (vagina/bladder)	97%	N/A
	31. >1 fistula tract	100%	N/A
	32. Anterior fistula opening	83%	N/A
Biological behavior	33. Fistula refractory to at least one surgical repair attempt	93%	N/A
	34. Recurrent fistulation (after initial healing)	93%	N/A
	35. Chronic fistula (ie non-healing)	100%	N/A
	36. Fistulae with a significant impact on patients QoL	77%	N/A
Associated anorectal lesions/disease	37. Proctitis	100%	N/A
	38. Perianal fissures	100%	N/A
	39. Anal stenosis	100%	N/A
	40. Presence of edematous skin tags	93%	N/A

Mostly due to agreement in the first Delphi round or due to merging/rephrasing in the second Delphi round of consensus.

^a^Defined as at least one perianal fistula intervention (including drainage of a perianal abscess or perianal surgery aimed at fistula closure) or abdominal bowel surgery (including amongst others an appendectomy or ileocoecal resection).

Abbreviations: N/A, not applicable; pCD, perianal Crohn’s disease.

### 3.2. The use of FCP as an adjunct for screening for CD

After 2 rounds, it was agreed that it is sensible to utilize FCP screening in all patients with any PAF (regardless of the complexity, biological behavior and co-existent perianal symptoms) to identify CD in an early phase (100%). In the first round of this Delphi study, participants were asked what cutoff value of FCP they would consider high enough to warrant further investigations to exclude CD in patients presenting with PAF, resulting in a median FCP cutoff of 150 mcg/g (IQR 150-200, 67%), which is consistent with the literature.^10^ Therefore, consensus was reached in the second round on an optimal cutoff value of FCP as a risk stratification tool of ≥150 mcg/g (92%).

### 3.3. The diagnostic work-up for CD in PAF patients with raised clinical suspicion on having CD

**Figure 1.**
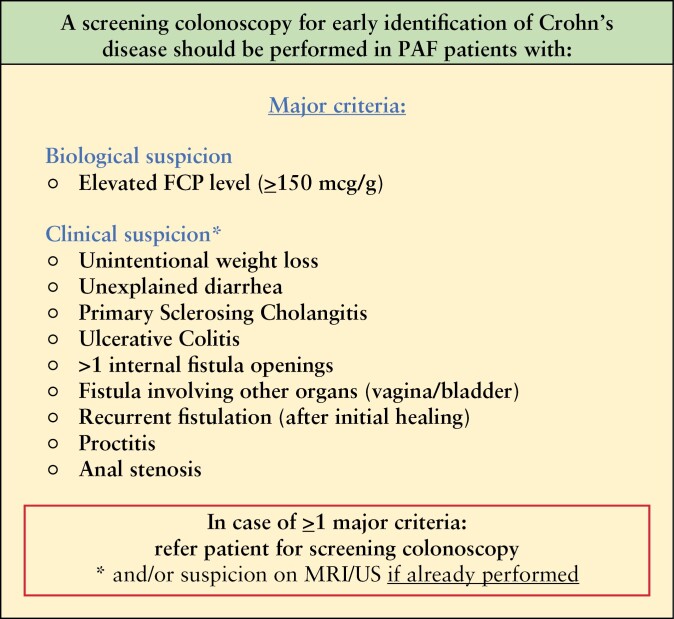
Algorithm on whom to perform a colonoscopy.

Consensus was reached within 2 rounds on performing a colonoscopy in PAF patients with elevated FCP levels and/or in case of clinical suspicion (92%). It was agreed that FCP should be included in the clinical decision tool as a major criterion. Ninety-four percent of participants agreed that a colonoscopy should not be performed in all PAF patients and that a scoring system when colonoscopy is indicated would be useful. In order to use the clinical decision tool in daily clinical practice, it was felt that stricter criteria for further screening (colonoscopy) were needed. Therefore, a third round was initiated in which all participants were asked to vote on which clinical (fistula) characteristics, independently of FCP, are sufficient to warrant a referral of a PAF patient for a colonoscopy. In total, 9 clinical (fistula) characteristics were selected as major criteria, defined as: unintentional weight loss (72%), unexplained diarrhea (75%), PSC (72%), UC (78%), >1 internal fistula openings (88%), fistula involving other organs (vagina/bladder, 88%), recurrent fistulation (after initial healing, 72%), proctitis (100%), and anal stenosis (88%). In Figure 1, a final algorithm on in whom to perform a colonoscopy is presented.

It was agreed that a colonoscopy should be based on current guidelines and, therefore, should include assessment of the terminal ileum and random biopsies of the ileum and colon (92%). Also, if colonoscopy is normal and clinical suspicion remains, patients should be referred for small bowel investigation (according to (local) guidelines) (100%). It was agreed that a patient can be diagnosed with a Crohn’s PAF based on having “any perianal fistula with associated luminal investigations showing radiological, endoscopic, or histologically confirmed luminal inflammation” (87%). In patients with a suspicion of CD in whom luminal disease has been excluded and cannot be categorized as isolated perianal CD (ipCD), it was advised to have standardized outpatient clinic follow-up in 1 year to re-assess the clinical decision tool on CD (88%). Advice on diagnosing and managing ipCD is presented in the associated paper on ipCD.

## 4. Discussion

In this Delphi study, international consensus was reached on a new, clinical decision tool including a practical and relevant algorithm for early identifying or excluding CD in patients with a PAF as a manifesting sign. Final consensus was reached on 12 statements, including screening of all PAF patients (regardless of the complexity, biological behavior, and co-existent perianal symptoms). In order to raise suspicion of CD in an early phase, PAF patients should be referred for a colonoscopy (including assessment of the terminal ileum and random biopsies of the ileum and colon) in case of elevated FCP levels (≥150 mcg/g) and/or in case of one clinical major criterion (defined as: unintentional weight loss, unexplained diarrhea, PSC, UC, > 1 internal fistula openings, fistula involving other organs (vagina/bladder), recurrent fistulation (after initial healing), proctitis, and anal stenosis). Also, clinical (fistula-)characteristics that warrant raised suspicion for CD were defined and in case of persistent suspicion of CD in patients in whom luminal disease has been excluded (and in case of no ipCD), it is advised to have standardized outpatient clinic follow-up in 1 year to re-assess the clinical decision tool on CD.

At the start of this Delphi study, 97% of all participants agreed that shortening the delay in diagnosis by using a clinical decision tool could improve outcomes in patients with perianal disease as a first symptom. However, only a minority of participants (33%) stated that they have a standardized protocol which they can use during daily clinical practice, underscoring the relevance of this project. In order to develop a generalizable, and simple, clinical decision tool it is important to focus on the so-called red flags as we did in this study. The consensus in this Delphi study on all red flags, suggestive of underlying CD, to be included in this decision tool is a significant step toward improved management of PAF patients.

Since it was recently shown that a prolonged time to CD diagnosis in patients with a PAF as a manifesting sign is associated with worse long-term outcomes,^7^ the question of in whom to perform diagnostic work-up for CD is a topic that merits consideration. Initially, it seems fair to subject all “suspected” patients presenting with perianal disease to further diagnostic work-up. However, it should be kept in mind that unnecessary colonoscopies should be avoided and noninvasive tools should help select patients with the highest risk of having CD. Therefore, a simple, practical, and relevant algorithm not only for finding but also for safely excluding CD in patients with PAF as a manifesting symptom is of the utmost importance.

In order to further enhance the potential diagnostic precision of the clinical decision tool, thereby avoiding unnecessary colonoscopies, consensus was reached on including FCP and 9 clinical (fistula) characteristics were identified as major criteria (eg, items that are independently sufficient to warrant a referral of a PAF patient for a colonoscopy). In this way, only patients with the highest risk of having CD will be referred for further diagnostic work-up, which conforms with the current European Crohn’s and Colitis Organisation (ECCO) guidelines concerning patients presenting with abdominal complaints.^15^ Since FCP has shown to be a potential and cost-effective biomarker in identifying patients with underlying CD when presenting with abdominal complaints,^11–14^ it is assumed that FCP could also play a major role in identifying patients at risk of having CD when presenting with a PAF.

A recent study by Chin Koon Siw et al. showed that approximately 25% of patients with recurrent PAF without signs of luminal disease on (ileo-)colonoscopy and/or computer tomography had evidence of small bowel inflammation on video capsule endoscopy.^16^ Therefore, it was agreed that patients should be referred for small bowel investigation (according to (local) guidelines) in case of persistent clinical suspicion after normal colonoscopy. After following our suggested algorithm, patients presenting with isolated perianal CD (ipCD) will remain. Isolated perianal CD does not fall within the scope of the project presented here. However, the studies of amongst others Pogacnik et al. and Schwartz et al. highlight the fact that approximately 5% of all patients have ipCD without any signs of luminal disease.^17,18^ Also, the role of anti-tumor necrosis factor (anti-TNF) in ipCD patients is a topic of interest.^19–21^ However, the best approach and specific guidelines on identifying and management of ipCD are currently lacking, on which we will elaborate in the second part of this Delphi study (ipCD).

One of the major strengths of this study is the wide global scope, which is emphasized by the diverse internationalities and expertise of the participating panel, reflecting a broad and international perspective on challenges associated with perianal manifestations of CD. In this Delphi study, a total of 30 experts participated, which is an adequate sample size according to among others Villiers et al, suggesting that an expert panel of more than thirty participants seldom improves outcomes.^22–25^ Finally, the literature states that the amount of questions and/or severity of questionnaires should be within limits in order to prevent dropout of participants,^26,27^ which was taken into account while developing the questionnaire.

This study has several limitations inherent to its design. At first, the majority of participating experts are affiliated with referral centers, which might hamper the external validity in daily clinical practice. However, this is inherent to the design of the standard Delphi methods, in which results are generated by experts in the field. Despite our efforts, we acknowledge that it is challenging to achieve a perfect global representation, which may influence the applicability of our clinical decision tool in all parts of the world. Another potential limitation to the widespread implementation of our new, clinical decision tool is that in some regions not all resources (eg, FCP measurement) are available or covered by health insurance, which may restrict access in countries with low resources. Also, FCP levels may vary across different patient populations (eg, elderly patients, patients with other inflammatory conditions, patients presenting with isolated ileal disease or patients presenting with isolated perianal disease without any signs of luminal disease), which should be kept in mind while assessing these values. It also should be kept in mind that the results of this study need to be validated in further prospective studies with longitudinal follow-up to determine the accuracy of this clinical tool to identify patients with luminal CD and that the optimal number of patients to screen in order to identify one patient at risk of having CD needs to be determined.

In conclusion, a clinical decision tool for early identification of CD in patients with a PAF as a first symptom could shorten delay in diagnosis and improve outcomes. International consensus for this tool, including a practical and relevant algorithm for identifying or excluding CD in patients with PAF as a manifesting sign, was reached within 3 rounds. Validation of our suggested clinical decision tool is crucial in order to confirm efficacy, generalizability, and practical utility.

## Supplementary Material

jjaf002_suppl_Supplementary_Table_S1

## Data Availability

The authors of the manuscript confirm that all data supporting the findings of this Delphi study are available within the manuscript. Supplementary details are available on reasonable request.
