# Supplementary material for: Diagnosing Crohn’s disease in presumed cryptoglandular perianal fistulas: an expert Delphi consensus on early identification of patients at risk of Crohn’s disease in perianal fistulas (PREFAB)
Source: J Crohns Colitis. 2025 Jan 7;19(1):jjaf002. doi: 10.1093/ecco-jcc/jjaf002 (PMC11783332; doi:10.1093/ecco-jcc/jjaf002)
Supplement: jjaf002_suppl_Supplementary_Table_S1 [file jjaf002_suppl_supplementary_table_s1.docx]

**Supplementary**

| **Name** | **Specialty** | **Affiliation** | **Practice environment** | **Country** | **Years of specialty experience** | **Publication/peer review experience in pCD/PAF** | **Gender** | **Race/ethnicity** |
| --- | --- | --- | --- | --- | --- | --- | --- | --- |
| Anders  Dige | Gastroenterologist | Department of Hepatology and Gastroenterology, Aarhus University Hospital | Specialist IBD referral centre | Denmark | 6 | Yes | Male | White (northern European) |
| Lilli  Lundby | Colorectal surgeon | Department of Surgery, Aarhus University Hospital | Specialist IBD & PAF referral centre | Denmark | 25 | Yes | Female | White (northern European) |
| Christianne J  Buskens | Colorectal surgeon  (Tertiary referral surgeon) | Department of Surgery, Amsterdam UMC, location VUmc, Amsterdam | Specialist IBD & PAF referral centre | Netherlands | 12 | Yes | Female | White (northern European) |
| Jaap  Stoker | Gastrointestinal radiologist | - Department of Radiology, Amsterdam UMC  - University of Amsterdam | Specialist IBD & PAF referral centre | Netherlands | 30 | Yes | Male | White (northern European) |
| Jarmila D W  van der Bilt | Colorectal surgeon | - Department of Surgery, Flevoziekenhuis, Almere  - Department of Surgery, Amsterdam UMC, location VUmc, Amsterdam | Specialist IBD & PAF referral centre | Netherlands | 13 | Yes | Female | White (northern European) |
| Dermot P B McGovern | Gastroenterologist | F. Widjaja Inflammatory Bowel Disease Institute, Cedars-Sinai Medical Center, Los Angeles | Specialist IBD referral centre | United States | 27 | Yes | Male | White (northern European) |
| Benjamin L Cohen | Gastroenterologist (IBD Clinical Lead) | Department of Gastroenterology, Hepatology, and Nutrition; Digestive Disease Institute, Cleveland Clinic, Ohio | Specialist IBD referral centre | United States | 14 | Yes | Male | White (northern European) |
| Stefan D  Holubar | Colorectal surgeon  (Director of Research, Colorectal Surgery/IBD Surgery) | Department of Colorectal Surgery, Digestive Diseases Institute, Cleveland Clinic, Ohio | Specialist IBD referral centre | United States | 14 | Yes | Male | White (northern European) |
| Nick  Powell | Gastroenterologist | Department of Digestion, Metabolism and Reproduction, Faculty of Medicine, Imperial College London | Specialist IBD referral centre | United Kingdom | 19 | Yes | Male | White (northern European) |
| Shaji  Sebastian | Gastroenterologist  (IBD service Lead) | IBD Unit, Department of Gastroenterology, Hull University Hospitals NHS Trust, Hull | Specialist IBD referral centre | United Kingdom | 20 | Yes | Male | Asian Indian |
| Antonino  Spinelli | Colorectal surgeon  (Tertiary referral surgeon) | - Department of Biomedical Sciences, Humanitas University, Pieve Emanuele, Milan  - IRCCS Humanitas Research Hospital, Rozzano, Milan | Specialist IBD referral centre | Italy | 23 | Yes | Male | White (Mediterranean) |
| Michele  Carvello | Colorectal surgeon  (Tertiary referral surgeon) | Humanitas clinical and research hospital, Rozzano, Milan | Specialist IBD referral centre | Italy | 10 | Yes | Male | White (Mediterranean) |
| Serre-Yu  Wong | Gastroenterologist | The Dr. Henry D. Janowitz Division of Gastroenterology, Icahn School of Medicine at Mount Sinai, New York city | Specialist IBD & PAF referral centre | United States | 7 | Yes | Female | Asian Chinese |
| Jean-Frédéric  Colombel | Gastroenterologist | The Dr. Henry D. Janowitz Division of Gastroenterology, Icahn School of Medicine at Mount Sinai, New York city | Specialist IBD & PAF referral centre | United States | 40 | Yes | Male | White (northern European) |
| Ignacio  Catalán-Serra | Gastroenterologist  (IBD Unit) | - Centre of Molecular Inflammation Research, Norwegian University of Science and Technology  - Department of Clinical and Molecular Medicine, Norwegian University of Science and Technology, Trondheim  - Gastroenterology, Department of Medicine, Levanger Hospital, Nord-Trøndelag Hospital Trust, Levanger | Non-specialist centre | Norway | 19 | No | Male | White (Mediterranean) |
| Susan J  Connor | Gastroenterologist  (IBD Service Lead) | - The University of New South Wales, Sydney  - Department of Gastroenterology, Liverpool Hospital, Sydney | Specialist IBD & PAF referral centre | Australia | 26 | Yes | Female | White (other) |
| Sulak  Anandabaskaran | Gastroenterologist | - The University of New South Wales, Sydney  - Department of Gastroenterology, Nepean Hospital, Sydney | Specialist IBD referral centre | Australia | 3 | Yes | Male | Asian Other |
| Jean-Frédéric  Leblanc | Gastroenterologist | Montreal Sacred Heart Hospital, University of Montreal, Montreal | Specialist IBD referral centre | Canada | 5 | Yes | Male | White (other) |
| Amy L  Lightner | Colorectal surgeon | Professor of Molecular Medicine and Colorectal Surgeon, Scripps Research Institute, La Jolla, California | Specialist IBD referral centre | United States | 10 | Yes | Female | White (northern European) |
| Ailsa L  Hart | Gastroenterologist | Department of Gastroenterology, St. Mark’s Hospital & Academic Institute | Specialist IBD & PAF referral centre | United Kingdom | 25 | Yes | Female | White (northern European) |
| Phil J  Tozer | Colorectal surgeon | Department of Surgery, St. Mark’s Hospital & Academic Institute | Specialist IBD & PAF referral centre | United Kingdom | 10 | Yes | Male | White (northern European) |
| Kapil  Sahnan | Colorectal surgeon | Department of Surgery, St. Mark’s Hospital & Academic Institute | Specialist IBD & PAF referral centre | United Kingdom | 2 | Yes | Male | Asian Indian |
| Philip F C  Lung | Gastrointestinal radiologist | Department of Radiology, St Mark’s Hospital & Academic Institute | Specialist IBD & PAF referral centre | United Kingdom | 13 | Yes | Male | Asian Chinese |
| Nik S  Ding | Gastroenterologist | Department of Gastroenterology, St. Vincent’s Hospital Melbourne | Specialist IBD & PAF referral centre | Australia | 15 | Yes | Male | Asian Chinese |
| Corina  Behrenbruch | Colorectal surgeon  (Tertiary referral colorectal surgeon) | Department of Surgery, St. Vincent’s Hospital Fitzroy Melbourne | Specialist IBD referral centre | Australia | 5 | Yes | Female | White (other) |
| Leon S  Winata | Gastrointestinal radiologist | Department of Radiology, St Vincent’s Hospital, Melbourne | Specialist IBD & PAF referral centre | Australia | 5 | Yes | Male | Asian Chinese |
| Jeffrey D  McCurdy | Gastroenterologist | - University of Ottawa, Department of Medicine, Division of Gastroenterology, Ottawa  - The Ottawa Hospital Research Institute, Ottawa | Specialist IBD referral centre | Canada | 12 | Yes | Male | White (other) |
| Jeroen  Geldof | Gastroenterologist | Department of Gastroenterology, University Hospital Ghent | Specialist IBD & PAF referral centre | Belgium | 5 | Yes | Male | White (northern European) |
| Danny  De Looze | Gastroenterologist | Department of Gastroenterology, University Hospital Ghent | Specialist IBD & PAF referral centre | Belgium | 32 | Yes | Male | White (northern European) |
| Isabelle  De Kock | Gastrointestinal radiologist | Department of Radiology, University Hospital Ghent | Specialist IBD & PAF referral centre | Belgium | 9 | Yes | Female | White (northern European) |
| Séverine  Vermeire | Gastroenterologist | - Department of Gastroenterology and Hepatology, Leuven University Hospitals, Leuven  - Department of Chronic Diseases and Metabolism, KU Leuven, Translational Research Center for Gastrointestinal Disorders (TARGID), Leuven | Specialist IBD & PAF referral centre | Belgium | 21 | Yes | Female | White (northern European) |
| Bram  Verstockt | Gastroenterologist | - Department of Gastroenterology and Hepatology, Leuven University Hospitals, Leuven  - Department of Chronic Diseases and Metabolism, KU Leuven, Translational Research Center for Gastrointestinal Disorders (TARGID), Leuven | Specialist IBD & PAF referral centre | Belgium | 4 | Yes | Male | White (northern European) |
| André  D’Hoore | Colorectal surgeon | Department of Abdominal Surgery, University Hospitals Leuven, Leuven | Specialist IBD referral centre | Belgium | 35 | Yes | Male | White (northern European) |
| Gabriele  Bislenghi | Colorectal surgeon | Department of Abdominal Surgery, University Hospitals Leuven, Leuven | Specialist IBD & PAF referral centre | Belgium | 10 | Yes | Male | White (Mediterranean) |
| David T  Rubin | Gastroenterologist | University of Chicago Medicine Inflammatory Bowel Disease Center, Chicago | Specialist IBD & PAF referral centre | United States | 24 | Yes | Male | White (other) |
| Benjamin D  McDonald | Gastroenterologist | University of Chicago Medicine Inflammatory Bowel Disease Center, Chicago | Specialist IBD & PAF referral centre | United States | 5 | Yes | Male | White (other) |
| Parakkal  Deepak | Gastroenterologist  (Site PI for all IBD Clinical Trials and related refractory case referrals) | Washington University School of Medicine in St. Louis, St. Louis, Missouri | Specialist IBD & PAF referral centre | United States | 8 | Yes | Male | Asian Indian |
| David H  Ballard | Abdominal radiologist | Mallinckrodt Institute of Radiology, Washington University St. Louis School of Medicine; St. Louis, Missouri | Specialist IBD referral centre | United States | 4 | Yes | Male | White (other) |
| Paulo G  Kotze | Colorectal surgeon | Health Sciences Postgraduate Program, Pontificia Universidade Catolica do Parana (PUCPR), Curitiba | Specialist IBD referral centre | Brazil | 27 | Yes | Male | White (other) |
| Carla B  Harmath | Radiologist  (Tertiary care-abdominal radiology) | University of Chicago Medicine Inflammatory Bowel Disease Center, Chicago | Specialist IBD & PAF referral centre | United States | 20+ | No | Female | Mixed race (other) |
| Sara  El Ouali | Gastroenterologist | Digestive Disease Institute, Cleveland Clinic Abu Dhabi | Specialist IBD & PAF referral centre | United Arab Emirates | 12 | Yes | Female | White (Mediterranean) |
| **First name** | **Specialty** | **Affiliation** | **Practice environment** | **Country** | **Years of specialty experience** | **Publication/peer review experience in pCD/PAF** | **Gender** | **Race/ethnicity** |
| Laurents P S  Stassen | Colorectal surgeon (lead of colorectal and oncologic surgery) | Department of Surgery, Maastricht University Medical Center; Nutrim Institute of Nutrition and Translational Research in Metabolism, Maastricht University | Specialist IBD & PAF referral centre | Netherlands | 32 | Yes | Male | White (northern European) |
| Froukje J  Hoogeboom | Colorectal surgeon  (tertiary referral IBD surgeon | Department of Surgery, University Medical Center Groningen | Specialist IBD & PAF referral centre | Netherlands | 12 | Yes | Female | White (northern European) |
| Marijn C  Visschedijk | Gastroenterologist (IBD expert gastroenterologist) | Department of Gastroenterology and Hepatology, University Medical Centre, Groningen | Specialist IBD & PAF referral centre | Netherlands | 10 | No | Female | White (northern European) |
| Koen W  van Dongen | Colorectal surgeon | Department of Surgery, Maasziekenhuis Pantein, Boxmeer | Specialist IBD & PAF referral centre | Netherlands | 12 | Yes | Male | White (northern European) |
| Marjolijn  Duijvestein | Gastroenterologist (tertiary IBD specialist) | Department of Gastroenterology and Hepatology, Radboud university medical center, Nijmegen | Specialist IBD & PAF referral centre | Netherlands | 9 | Yes | Female | White (northern European) |
| Oddeke  van Ruler | Colorectal surgeon  (tertiary referral IBD/PAF surgeon) | - Department of Surgery, IJsselland Hospital  - Department of Surgery, Erasmus Medical Center | Specialist IBD & perianal fistula referral centre | Netherlands | 12 | Yes | Female | White (northerm European) |
| Koen C M J  Peeters | Colorectal surgeon | Department of Surgery, Leiden University Medical Center | Specialist IBD & PAF referral centre | Netherlands | 20 | Yes | Male | White (northerm European) |
| Andrea E  van der Meulen-de Jong | Gastroenterologist  (Tertiary IBD gastroenterologist) | Department of Gastroenterology and Hepatology, Leiden University Medical Center | Specialist IBD & PAF referral centre | Netherlands | 13 | Yes | Female | White (northerm European) |
| Milan C  Richir | Colorectal surgeon (tertiary referral IBD surgeon) | Department of Surgery, University Medical Center Utrecht | Specialist IBD referral centre | Netherlands | 10 | Yes | Male | White (northern European) |
| Fiona D M  van Schaik | Gastroenterologist | Department of Gastroenterology and Hepatology, University Medical Center Utrecht | Specialist IBD referral centre | Netherlands | 10 | No | Female | White (northern European) |
| Marie J  Pierik | Gastroenterologist (IBD service lead) | Department of gastroenterology-hepatology, School for Nutrition and Translational Research in Metabolism (NUTRIM), Maastricht University Medical Centre+, Maastricht | Specialist IBD & PAF referral centre | Netherlands | 18 | Yes | Female | White (northern European) |
| Marco W  Mundt | Gastroenterologist | Department of Gastroenterology and Hepatology, Flevoziekenhuis, Almere | Specialist IBD referral centre | Netherlands | 20 | Yes | Male | White (northern European) |
| David D E  Zimmerman | Colorectal surgeon (EBSQ Coloproctology certified) | Fistula Expertise Center, ETZ, Tilburg | Specialist PAF referral centre | Netherlands | 15 | Yes | Male | White (northern European) |
| Ingrid J M  Han-Geurts | Colorectal surgeon | Proctos Clinics | Specialist PAF referral centre | Netherlands | 23 | No | Female | White (northern European) |

**Supplementary Table 1** Details of all investigators/expert panel members (in no particular order)
